# Medical applicant general practice experience and career aspirations: a questionnaire study

**DOI:** 10.3399/BJGPO.2021.0023

**Published:** 2021-04-14

**Authors:** Priyesh Agravat, Tafsir Ahmed, Esme Goudie, Shahraz Islam, Douglas GJ McKechnie, Haji Mohamed Abdirahman, Mahnoor Ahmed, Amer Al-Balah, Ayesha Alam, Fahima Amin, Sara Beqiri, Smruthy Chakka, Katy Chisenga, Roshni Goodka, Nida Hafiz, Ankita Kotamarthi, Ayobami Emmanuel Olatunji, Molly V Fyfe, Nina Dutta, Ian Chris McManus, David Harrison, Katherine Woolf

**Affiliations:** 1 University College London Medical School, London, UK; 2 Department of Primary Care and Population Health, University College London, London, UK; 3 Department of Primary Care and Public Health, Imperial College London, London, UK; 4 Brighton and Sussex University Hospitals NHS Trust, Brighton, UK

**Keywords:** Career choice, general practitioners, socioeconomic factors, schools, education, premedical

## Abstract

**Background:**

Increasing access to general practice work experience placements for school students is a strategy for improving general practice recruitment, despite limited evidence and concerns surrounding equity of access to general practice experiences.

**Aims:**

To examine the association between undertaking general practice experience and the perceptions of general practice as an appealing future career among prospective medical applicants. To identify socioeconomic factors associated with obtaining general practice experience.

**Design & setting:**

Cross-sectional questionnaire study in the UK.

**Method:**

Participants were UK residents aged ≥16 years and seriously considering applying to study medicine in 2019/2020. They were invited to take part via the University Clinical Aptitude Test (UCAT). Questionnaire data were analysed using a linear regression of general practice appeal on general practice experience, adjusting for career motivations and demographics, and a logistic regression of general practice experience on measures of social capital and demographics.

**Results:**

Of 6391 responders, 4031 were in their last year of school. General practice experience predicted general practice appeal after adjusting for career motivation and demographics (b = 0.37, standard error [SE] = 0.06, *P*<0.00001). General practice experience was more common among students at private (odds ratio [OR] = 1.65, 95% confidence interval [CI] = 1.31 to 2.08, *P*<0.0001) or grammar schools (OR = 1.33, 95% CI = 1.02 to 1.72, *P* = 0.03) and in the highest socioeconomic group (OR = 1.62, 95% CI = 1.28 to 2.05, *P*<0.0001), and less likely among students of ‘other’ ethnicity (OR = 0.37, 95% CI = 0.20 to 0.67, *P* = 0.0011).

**Conclusion:**

Having general practice experience prior to medical school was associated with finding general practice appealing, which supports its utility in recruitment. Applicants from more deprived backgrounds were less likely to have had a general practice experience, possibly through lack of accessible opportunities.

## How this fits in

There is very limited evidence about the effectiveness of general practice work experience as a potential recruitment strategy, and concerns about equity of access. In a large cross-sectional study of 4031 prospective medical applicants, exposure to general practice was associated with a higher appeal of general practice as a future career, after adjusting for motivation to study medicine and their demographics. Attending a private or grammar school and being in the highest socioeconomic group increased the odds of having had general practice experience, while being in the Arab or 'other' ethnic group, from lower socioeconomic groups, and attending a state school decreased the odds of having a general practice experience. Offering general practice experiences to prospective medical applicants may help encourage intentions for a career in general practice, but increased efforts are needed to ensure equity of access.

## Introduction

There is an ongoing workforce crisis in general practice. One recommendation of the 2016 Wass report^1^ was to increase provision of, and access to, work experience placements in general practice for medical applicants, ultimately aiming to improve postgraduate general practice recruitment.

The UK Medical Schools Council (MSC) describes work experience as *‘* *an essential part of the medical school application* *’*;^2^ their definition of work experience includes paid employment, volunteering (for example, caring roles), and direct observation of health care, such as ‘shadowing’ a healthcare professional. This remains the case during the COVID-19 pandemic, although medical schools recognise the limited opportunities students have to gain work experience, and MSC recommends two virtual work experience courses (one focused on general practice), as well as volunteering and keeping a reflective diary.^3^

While the role of general practice exposure in medical school has been analysed in detail^4,5^ there is little research examining the influences on interest in general practice careers among medical applicants, including the role of work experience. Two single-institution studies support a positive effect of work experience on motivation and medical career choice, though they also report negative experiences including difficulty in arranging general practice experience.^6,7^ In both, applicants and students were more likely to have done work experience in a hospital than in general practice. Use of social connections with healthcare professionals to obtain work experience was reported, and those from fee-paying schools received more support. Generally, obtaining healthcare work experience seems more challenging for students from under-represented backgrounds, such as students at non-selective state schools and students from lower socioeconomic backgrounds.

The UK Medical Applicant Cohort Study (UKMACS) is a longitudinal questionnaire study of prospective medical school applicants, run by a study group at University College London, and recruiting primarily from UCAT registrars in 2019.^8^ Almost all UK medical applicants sit the UCAT (98% did so in an analysis of 22 520 school-aged applicants between 2010 and 2014^9^). This study used UKMACS data to answer two research questions:

1. Is the appeal of general practice as a future career associated with having a general practice experience after adjusting for possible confounders?
2. Which social and demographic features are associated with having a GP experience?

## Method

### Study design and participant recruitment

This study is a cross-sectional questionnaire study using Wave 1 UKMACS data (see Supplementary Appendix S1 for questionnaire).

Those aged ≥16 years, resident in the UK or Crown dependencies, and considering applying for medicine in the UK during the 2019/2020 application period were eligible.

All registrars for the UCAT who consented (*n* = 18 359) were invited, via email, to complete a questionnaire between 1 May 2019 and 15 October 2019 (the medical school application deadline). The survey was advertised widely including on the Biomedical Admissions Test and MSC websites, several medical school widening participation events and open days, and on social media.

The UCAT is also used for dental and veterinary degree admissions; responders were asked to only complete the survey if they were considering applying for medicine.

This study limited the analysis to those born after 31 August 2001, who would mostly be in their last year of school on 15 October 2019, to exclude older applicants who may have had more opportunities and/or time to gain experience.

### Study variables

#### Main variables

The two main study variables were general practice appeal and general practice experience.

General practice appeal was derived from a question in which participants were asked to rate how appealing they found 13 specialty/medical careers (1 = very unappealing, 2 = moderately unappealing, 3 = uncertain, 4 = moderately appealing, and 5 = very appealing).

General practice experience was derived from a free-text question asking responders to describe *‘all the courses or activities you have done that are relevant to applying to medicine, such as access to university programmes, widening participation/outreach programmes, summer schools, taster days, or work experience* *’*. Twenty medical students, doctors, and staff coded the free-text experience responses. The coding strategy and coding framework are detailed in Supplementary Appendix S2. Briefly, experiences were categorised by type of activity (for example, work experience, course, or lecture), whether the activity was specific to medicine, and its location (for example, hospital, GP surgery, or university). General practice experience included any medical experience (including work experience, volunteering, or shadowing) in a GP surgery or primary care setting in which GPs work (for example, a walk-in centre). ‘Hospital experience’ incorporated any medicine experience in a hospital. The number of experiences was the sum of all experiences of any type in any location.

### Background variables

#### Sociodemographic and education variables

The following variables were collected from the questionnaire: sex; ethnicity (using the 2011 UK census groups); whether responders had at least one parent/carer in the highest socioeconomic group; whether responders had at least one parent/carer who was a doctor; the approximate number of doctors or medical students who responders knew socially; the approximate number of people responders knew who were considering applying to medical school; school type (state, grammar, or private); and mean GCSE grades. Index of Multiple Deprivation quintile was derived from home postcode, which was provided by UCAT.

#### Psychological variables

Questionnaire items included motivation for becoming a doctor and appeal of working as a doctor in different geographical and socioeconomic settings (both adapted from McManus *et al*,^10^ in which they were associated with specialty preference), and ‘big five’ personality traits (from *Understanding Society* ^11^).

#### Statistical analysis

The data were explored using descriptive statistics and univariate analyses in IBM SPSS Statistics (version 26). Multivariate analyses were performed in R (version 4.0.02), with multiple imputation of missing values using the mice package.^12^ The full list of variables imputed is included in Supplementary Table S1. Missing values were calculated using predictive mean matching, whereby imputed values were taken from the range of values in the data. The number of imputations was set at 10. Regression analyses were carried out on the 10 imputed mira datasets using the ‘lm’ function within the ‘with’ function; then the sets of results in the mipo dataset were combined with the ‘pool’ function. *P* values were Bonferroni-corrected for multiple comparisons.

To answer the first research question, a linear regression of general practice appeal on general practice experience (a binary variable: none [0] versus at least one [1]) was performed, controlling for potential confounders including sociodemographic, education, and psychological variables. To answer the second research question, a logistic regression of general practice experience on sociodemographic and education variables was performed.

## Results

### Participants

A total of 6391 responders completed the survey. Of the 4160 born after 31 August 2001, 129 were missing postcode data or did not have a valid UK postcode of residence and therefore did not meet the inclusion criteria, leaving 4031 for analysis. See Supplementary Table S2 for full descriptive statistics.

#### Application experiences

Most participants (*n* = 3737, 92.7%) reported at least one application experience. Only 16.7% (*n* = 673) of participants reported a general practice medical experience (89.0% of these experiences were work experience). By contrast, over half (57.1%, *n* = 2302) of participants reported a hospital medical experience. Of those with a general practice experience, two-thirds (68.5%, *n* = 461) also had a hospital experience. Having a general practice experience but no hospital experience was rare: only 5.3% of all participants (*n* = 212).

#### Appeal of general practice and other specialties

General practice was the sixth most appealing specialty (mean = 3.27, standard deviation [SD] = 1.44). The most appealing were internal medicine (mean = 4.32, SD = 0.91) and surgery (mean = 4.16, SD = 1.19). See Supplementary Table S3.

#### Education

Most participants (67.4%, *n* = 2638) attended a state school. Similar numbers attended private (16.4%, *n* = 641) and grammar schools (16.2%, *n* = 635).

GCSE point scores were self-reported by 92.2% (*n* = 3716) of participants. Scores were normally distributed with a mean of 77.5 (SD = 15.7). Participants took an average of 10 GCSEs, making the mean GCSE points score 7.5 (SD = 0.9).

#### Sociodemographic and social capital factors

Two-thirds of participants (68.0%, *n* = 2635) had at least one parent/carer in the highest socioeconomic group of professionals/senior managers; 10.8% (*n* = 437) had at least one doctor parent/carer.

Deprivation was calculated for 90.5% (*n* = 3648) of participants with home postcodes in England, Scotland, and Wales. Participants were reasonably evenly distributed across deciles, except that the least deprived decile contained 14.5% of participants.

Participants knew, on average, six to ten other medical applicants, and only 3.2% (*n* = 128) knew no other applicants. They knew, on average, two to five people training or working as doctors, with 27.9% (*n* = 1124) of participants knowing no medics.

### Univariate analyses

#### Relationship between general practice appeal and general practice experience

Participants with no general practice experiences found general practice less appealing (mean = 3.22, SD = 1.45) than those with ≥1 GP experiences (mean = 3.51, SD = 1.43) (t [3.951] = –4.65; *P*<0.001).

**Figure 1.**
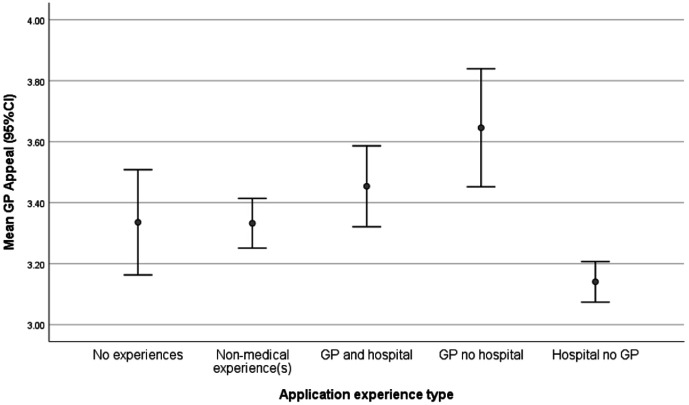
Mean general practice appeal by experience type. GP = general practice.

Participants with a general practice experience and no hospital experience were most likely to find general practice appealing (mean = 3.64, SD = 1.41), whereas those with hospital and no general practice experience were the least likely to find general practice appealing (mean = 3.14, SD = 1.44) (F [4.3952] = 9.69, *P*<0.001) (Figure 1).

#### Relationship between general practice appeal and background factors

Higher general practice appeal was correlated with wanting to practise medicine close to home (*r* = 0.173, *P*<0.001), in the countryside (*r* = 0.129, *P*<0.001), and not overseas (*r* = −0.108, *P*<0.001). There were smaller (*r*<0.1) but highly significant (*P*<0.001) positive correlations between general practice appeal and the ‘big five’ personality factor, agreeableness; being of Asian ethnicity; attending a state school; lower GCSE points; wanting to be a doctor to be helpful to society; having an occupation that was economically secure, but not highly pressurised or with a requirement for travel; wanting to practise in the NHS; and practise privately, but not in a city or in the armed forces (see Supplementary Table S4).

#### Relationship between general practice experience and background factors

General practice experience was positively correlated with attending private school (*r* = 0.100, *P*<0.001), having a parent in the highest socioeconomic group (*r* = 0.10, *P*<0.001), having a doctor parent (*r* = 0.072, *P*<0.001), knowing more doctors (*r* = 0.097, *P*<0.001) and medical applicants (*r* = 0.061, *P*<0.001), living in a less deprived postcode (*r* = 0.056, *P*<0.001), higher GCSE points (*r* = 0.086, *P*<0.001), and being of White ethnicity, rather than in the ’other’ ethnic group (*r* = 0.05, *P* = 0.002). It was also correlated with having more experiences of any type (*r* = 0.26, *P*<0.001), and with having a hospital experience (*r* = 0.10, *P*<0.001) (see Supplementary Table S5).

### Multivariate analyses

#### The appeal of general practice as a future career

Regressing general practice experience onto general practice appeal in a simple linear regression showed that having a general practice experience was a small but highly statistically significant predictor of finding general practice appealing (b = 0.28, SE = 0.06, *P*<0.00001), meaning those with a general practice experience scored 0.28 points higher general practice appeal compared to those without.

In the full model (Table 1) general practice experience remained one of the most highly significant predictors of general practice appeal (b = 0.37, SE = 0.06, *P*<0.00001), with the effect being slightly larger. Variance inflation factor statistics for the predictors were all between 1.0 and 1.4, suggesting this increase was unlikely to be due to multicollinearity, and the multiple imputation of missing values means the effect could not be a result of a reduction in sample size due to listwise deletion of missing values. It seems instead that adjusting for background factors did indeed slightly increase the size of the relationship.

**Table 1.** Predictors of finding general practice appealing as a future career (pooled results from 10 multiple imputations). Predictors ordered by significance level

Predictor^a^	Unstandardised estimate (b)	SE	Lower 95% CI	Upper 95% CI	*P* value
Intercept	2.060	0.392	1.292	2.827	<0.00001
Wanting to practice in rural location	0.153	0.021	0.111	0.194	<0.00001^b^
Wanting to practice close to where they live or grew up	0.128	0.02	0.089	0.168	<0.00001^b^
Has had at least one GP experience	0.365	0.061	0.245	0.485	<0.00001^b^
Wanting to practice overseas	–0.107	0.022	–0.150	–0.065	<0.00001^b^
Wanting to practice in the NHS	0.133	0.028	0.077	0.189	<0.00001^b^
Personality trait: agreeable	0.131	0.028	0.075	0.187	<0.00001^b^
Wanting to practice in the armed forces	–0.077	0.017	–0.111	–0.042	0.00001^b^
Wanting to practice in the private sector	0.090	0.022	0.047	0.133	0.00004^b^
Motivated by an economically secure occupation	0.099	0.026	0.049	0.150	0.00011^b^
Motivated by a challenging role under pressure	–0.098	0.026	–0.150	–0.047	0.00019^b^
Wanting to practice in an urban area	–0.11	0.03	–0.169	–0.050	0.00032^b^
Motivated by being helpful to others and society	0.159	0.049	0.063	0.255	0.00116^b^
Asian ethnicity	0.165	0.054	0.059	0.270	0.0022
Has had at least one hospital experience	–0.146	0.048	–0.240	–0.051	0.00247
GCSE points	–0.005	0.002	–0.008	–0.001	0.00705
Personality trait: conscientious	–0.075	0.028	–0.131	–0.020	0.00782
Wanting to practice in a deprived area	0.063	0.024	0.016	0.110	0.00846
Motivated by expressing own values and interests	–0.062	0.026	–0.112	–0.012	0.01487
'Other' ethnicity^c^	0.272	0.113	0.050	0.493	0.01643
Personality trait: neurotic	–0.042	0.018	–0.079	–0.006	0.02197
Attends a private school	–0.145	0.065	–0.273	–0.018	0.0259
Personality trait: extraverted	–0.042	0.019	–0.080	–0.004	0.03175
Black ethnicity	0.151	0.086	–0.017	0.319	0.07784
Wanting to practice in an affluent area	0.044	0.029	–0.012	0.100	0.12622
Attends a grammar school	–0.096	0.069	–0.231	0.040	0.16673
Motivated by opportunities to travel	–0.011	0.023	–0.056	0.033	0.62023
Mixed ethnicity	0.039	0.106	–0.169	0.247	0.71371
Total number of experiences related to medicine	–0.002	0.012	–0.025	0.021	0.88662
Personality trait: open	–0.002	0.022	–0.044	0.041	0.94475

^a^Reference category for ethnic groups = White ethnic group; reference category for school type = state-funded school. ^b^Bonferroni corrected significance level = 0.0017. ^c^Includes Arab ethnicity. SE = standard error.

#### Having general practice experience

A logistic regression showed participants who attended a private school (OR = 1.65, 95% CI = 1.31 to 2.08, *P*<0.0001) or a grammar school (OR = 1.33, 95% CI = 1.02 to 1.72, *P* = 0.03), and who had at least one parent in the highest socioeconomic group (OR = 1.62, 95% CI = 1.28 to 2.05, *P*<0.0001) were more likely to have a general practice experience. Having more application experiences in total was also a significant predictor of general practice experience (OR = 1.36, 95% CI = 1.30 to 1.41, *P*<0.0001). Participants in the 'other' ethnic group were less likely than White participants to have a general practice experience (OR = 0.37, 95% CI = 0.20 to 0.67, *P* = 0.001) (Table 2).

**Table 2.** Predictors of having at least one GP experience (pooled results from 10 multiple imputations). Predictors ordered by significance level

Predictor^a^	Unstandardised estimate (b)	SE	OR	Lower 95% CI	Upper 95% CI	*P* value
Total number of experiences relating to medicine	0.30	0.02	1.36	1.30	1.41	<0.0001^b^
Attends a private school	0.50	0.12	1.65	1.31	2.08	<0.0001^b^
Has at least one parent/carer in ‘professional’ socioeconomic group	0.48	0.12	1.62	1.28	2.05	0.0001^b^
'Other' ethnicity^c^	–0.99	0.31	0.37	0.20	0.67	0.0011^b^
Attends a grammar school	0.28	0.13	1.33	1.02	1.72	0.0321^b^
Mixed ethnicity	–0.42	0.24	0.66	0.41	1.05	0.0779
Has at least one parent/carer who is a doctor	0.23	0.14	1.26	0.96	1.66	0.0948
Number of medical students or doctors the applicant knows	0.07	0.04	1.07	0.99	1.16	0.098
Mean GCSE points	0.01	0.01	1.01	1.00	1.01	0.1263
Black ethnicity	–0.27	0.18	0.76	0.53	1.08	0.1285
Asian ethnicity	–0.05	0.11	0.95	0.77	1.18	0.6525
Has had at least one hospital medicine experience	0.03	0.10	1.03	0.85	1.25	0.7787
Lives in a less deprived postcode	–0.01	0.02	1.00	0.96	1.03	0.851
Number of other applicants to medicine that the applicant knows	0.0007	0.04	1.00	0.92	1.08	0.9853

^a^Reference category for ethnic groups is the White ethnic group; reference category for school type is state-funded school. ^b^Predictors statistically significant at the Bonferroni corrected level = 0.0035. ^c^Includes Arab ethnicity. OR = odds ratio. SE = standard error.

## Discussion

### Summary

To the authors' knowledge, this is the first large-scale quantitative study of the relationship between pre-medical school GP experiences and the appeal of general practice in people interested in applying to medicine. Participants were over three times more likely to have had an experience in hospital than in general practice. General practice experience had a small but statistically significant, positive association with appeal of general practice as a future career choice, after controlling for confounders. Students from a state school, without a parent in the highest socioeconomic group, and of 'other' ethnicity (including the Arab ethnic group) were less likely to have had general practice experience, which suggests access difficulties.

### Strengths and limitations

The large sample size includes diverse students from across the entire UK. To the authors' knowledge, this is the only large-scale study of data on pre-medical school experiences. This study analysed all participant experiences in general practice together, which was mostly self-described ‘work experience’, but also included ‘shadowing’ and ‘volunteering’, the exact nature of which will depend on participants’ own definitions of those terms. This range of experiences likely differ in length, structure, quality, and nature. Some might have been negative experiences. This may diminish the strength of association between general practice experiences and general practice interest, meaning that these results may underestimate any true effect of positive general practice experiences on career aspirations. This study is also unable to determine which features of general practice experience are most strongly linked with interest in general practice. This analysis does not take into account the availability of general practice experience, which may vary by geographic location.

This study cannot assume that having a general practice experience causes applicants to find general practice more appealing — it may be that students who are already interested in general practice were more likely to seek out general practice work experience placements. In trying to understand whether lack of general practice experience is due to lack of interest or lack of access, it can be useful to consider hospital and other experiences. Indeed, this analysis of the social capital predictors of general practice experiences included hospital experience(s) and total number of experiences as potential predictors, with results showing that participants without a parent in the highest socioeconomic group, those in the ‘other’ ethnic group (versus White), and those at state schools (versus private or grammar schools), were all less likely to have general practice experiences, regardless of whether or not they had hospital experience, and regardless of whether they had a lot or few experiences overall. Moreover, attending a private school (versus state school) was negatively correlated with finding general practice appealing, yet was a positive predictor of having a general practice experience, and there was no significant relationship between parental socioeconomic group and general practice appeal, regardless of the number of hospital experiences. Taken together, this makes it feasible that the relative lack of general practice experiences in participants with less social capital could be due to lack of access rather than lack of interest, and therefore access may be problematic for students from more deprived backgrounds interested in general practice.

Career aspirations and interests are complex and can be influenced by many factors throughout life. Other unmeasured factors (for example, the portrayal of doctors in popular culture) likely influence how appealing the participants in this study found general practice, and appeal at this stage may not predict general practice career choice years later.

### Comparison with existing literature

This study's findings accord with those showing positive exposure to general practice is associated with career interest in the specialty, though almost all of this was done at undergraduate^4^ or postgraduate level^13,14^ rather than prior to university entry, adding novelty to this study's findings.

Fewer students had a general practice experience compared to a hospital experience in this sample, which replicates prior findings;^6,7^ however, the proportion of students in this study who had a general practice experience was much lower (16.7% versus 44.6%, from Nicholls *et al* ^6^). Those previous studies recruited medical applicants and students, whereas this study includes those interested in applying to medicine who might not apply or who have applied unsuccessfully. These groups might be less likely to have had a general practice experience than those who committed to applying or got in. Alternatively, the medical schools in which the previous studies were conducted may have been more likely to attract or select students with general practice experience; indeed, one was operating a widening participation programme focusing on supporting general practice work experience placements.^6^

General practice work experience can be challenging to obtain, perhaps due to a relative paucity of organised work experience schemes compared to hospitals. Previous studies have found students at fee-paying schools and in higher socioeconomic groups, especially with medical parents, may find it easier to access medical work experience.^6,15,16^ This study's findings concur that parental socioeconomic status, but not having a medical parent or knowing doctors socially, was an independent predictor of general practice experience. The authors are not aware of research specifically describing a relationship between ethnicity and obtaining medical work experience.

### Implications for research and practice

These results support increased access to pre-medical school general practice experience as a general practice recruitment strategy. Further research will follow-up those participants who entered medical school using the UK Medical Education Database^17^ to determine the longitudinal effects of general practice experience. Longitudinal studies of the medical career interests of younger school students could help to determine the direction of causality between general practice appeal and general practice experience.^18^

This study's data (Figure 1) suggest that participants who found general practice most appealing were those with general practice experience but no hospital experience, whereas those who found it least appealing were those with hospital experience and no general practice experience. This finding requires replication, particularly given the small proportion of participants with general practice but no hospital experience, but it could mean that those strongly interested in general practice pre-application seek out general practice experience above hospital experience, or that hospital experience perhaps puts applicants off general practice.

Hospital work experience remains more common than general practice work experience; access to the latter could be improved in line with the former, and general practice work experience could be better promoted. The Royal College of General Practitioners recently launched an online general practice work experience platform, Observe GP, to increase access.^19^ At the time of writing, it had over 10 000 sign-ups and was being evaluated. Practices, perhaps working in networks or federations, could facilitate access to work experience by designing and publicising official work experience schemes, as many hospitals do,^20–22^ and by affiliating with widening participation schemes at university, some of which receive funding from Health Education England. The Royal College of General Practitioners also publish resources to support practices who are interested in hosting work experience students.^23^

Stakeholders should consider the potential impact of socioeconomic status, schooling, and ethnicity on the ease of attaining general practice work experience and should aim to ensure that under-represented groups of applicants are supported to do so; this would support the goal of promoting a diverse general practice workforce. This is unlikely to be limited to general practice experiences^24^ and so the authors suggest that this needs to be a ‘cross-specialty’ conversation,^25^ and future research between social capital and pre-medical school experiences in all medical settings is needed.

This study demonstrated that, in a large sample of UK-based students interested in applying to medicine, experience in general practice is positively associated with appeal of general practice as a future career. Providing work experience opportunities for medical applicants may be a valid strategy to improve recruitment. However, general practice experience was much less common than hospital-based experience. There was evidence to suggest those from lower socioeconomic groups and at non-selective state schools were less likely to have accessed general practice experience. Equity of access could be prioritised.
